# 

**Published:** 1848-04

**Authors:** 


CONTENTS,

OF THE

### DENTAL REGISTER OF THE WEST.

### APRIL NO., 1848.

Art. 1. Dr. Slack's Address, ........ Page 115
" 2. Facts for the People, by H. Crane, D. D. S., ........ 132
" 3. Comments on the action of the American Society of Dental Surgeons, By B. B. Brown, M. D. D. S., St. L. Ed. ........ 134
" 4. Filing Teeth, by Dr. Taylor, Cin. Ed. ........ 142
" 5. Death from the Inhalation of Chloroform, ........ 148
" 6. A whole Medical Staff Anæstheticised, ........ 152
" 7. Letter from Dr. Berry, ........ 153
" 8. Furnace, by Dr. Hoy, ........ 154
" 9. Destroying, vs. Capping the Nerve of Human Teeth, by Dr. Whitney 156
" 10. Excessive Hœmorrhage from extracting a tooth, ........ 158
" 11. Harris's Principles and Practice of Dental Surgery, by Cin. Ed., ........ 160
" 12. Professional Jealousy, by J. Allen, ........ 164
" 13. Gen'l Tom Thumb, by Cin. Ed., ........ 166
" 14. Ohio College of Dental Surgery, by Cin. Ed., ........ 168
" 15. Prof's Cook and Rogers, by Cin. Ed., ........ 169
" 16. Ether and Chloroform, by Cin. Ed., ........ 169
" 17. Ambler's Journal of Dental Operations for 1848, by Cin. Ed., ........ 170
" 18. Subscribers, by Cin. Ed., ........ 170
" 19. Dr. Ide, by Cin. Ed., ........ 170
" 20. Dr. Taylor's Essays on Filling Teeth, by Cin. Ed., ........ 170

